# Age Estimates for the Buckwheat Family Polygonaceae Based on Sequence Data Calibrated by Fossils and with a Focus on the Amphi-Pacific *Muehlenbeckia*

**DOI:** 10.1371/journal.pone.0061261

**Published:** 2013-04-05

**Authors:** Tanja M. Schuster, Sabrina D. Setaro, Kathleen A. Kron

**Affiliations:** Department of Biology, Wake Forest University, Winston-Salem, North Carolina, United States of America; University of Sydney, Australia

## Abstract

The buckwheat family Polygonaceae is a diverse group of plants and is a good model for investigating biogeography, breeding systems, coevolution with symbionts such as ants and fungi, functional trait evolution, hybridization, invasiveness, morphological plasticity, pollen morphology and wood anatomy. The main goal of this study was to obtain age estimates for Polygonaceae by calibrating a Bayesian phylogenetic analysis, using a relaxed molecular clock with fossil data. Based on the age estimates, we also develop hypotheses about the historical biogeography of the Southern Hemisphere group *Muehlenbeckia*. We are interested in addressing whether vicariance or dispersal could account for the diversification of *Muehlenbeckia*, which has a “Gondwanan” distribution.

Eighty-one species of Polygonaceae were analysed with MrBayes to infer species relationships. One nuclear (nrITS) and three chloroplast markers (the *trn*L-*trn*F spacer region, *mat*K and *ndh*F genes) were used. The molecular data were also analysed with Beast to estimate divergence times. Seven calibration points including fossil pollen and a leaf fossil of *Muehlenbeckia* were used to infer node ages.

Results of the Beast analyses indicate an age of 110.9 (exponential/lognormal priors)/118.7 (uniform priors) million years (Myr) with an uncertainty interval of (90.7–125.0) Myr for the stem age of Polygonaceae. This age is older than previously thought (Maastrichtian, approximately 65.5–70.6 Myr). The estimated divergence time for *Muehlenbeckia* is 41.0/41.6 (39.6–47.8) Myr and its crown clade is 20.5/22.3 (14.2–33.5) Myr old. Because the breakup of Gondwana occurred from 95–30 Myr ago, diversification of *Muehlenbeckia* is best explained by oceanic long-distance and maybe stepping-stone dispersal rather than vicariance. This study is the first to give age estimates for clades of Polygonaceae and functions as a jumping-off point for future studies on the historical biogeography of the family.

## Introduction

The buckwheat family Polygonaceae Juss. with approximately 1,200 species [1] is morphologically diverse and growth forms include trees, shrubs, vines, lianas and herbs. Polygonaceae are found in a wide range of habitats from the Arctic to the tropics, from montane to lowland regions, and from arid to aquatic situations. Many thrive in disturbed habitats, are primary successors or short-lived fire ephemerals. Few synapomorphies exist for the entire group, but ocreae are found in most species. Ocreae are located at the leaf base and they encircle and sheathe the stem at the node, which may be swollen. Pollen and wood are exceptionally diverse in the buckwheat family and should be investigated with cladistic studies. Even though Polygonaceae are not considered a group of great economic value, some species are used as crops and in horticulture. For example, buckwheat (*Fagopyrum esculentum* Moench) is a staple in Russia and soba noodles made from its flour are popular in Japanese cuisine. Several species, such as *Antigonon leptopus* Hook & Arn., *Persicaria perfoliata* (L.) H.Gross and *Reynoutria japonica* Houtt. are used as ornamentals and have become invasive outside their native range.

Polygonaceae are mainly distributed in north temperate regions [2]. For example, 35 of the 50 described genera occur in North America [2] and Polygonaceae are one of the approximately 30 plant families that occur north of the Arctic Circle [3]. Few members of Polygonaceae are native to the Southern Hemisphere and these include *Afrobrunnichia* Hutch. & Dalziel, *Chorizanthe* R.Br. ex Benth., *Coccoloba* P.Browne, *Duma* T.M.Schust., *Emex* Neck. ex Campd., *Magoniella* Sanchez and Reveal, *Muehlenbeckia* Meisn., *Oxygonum* Burch. ex Campd., *Persicaria* Mill., *Polygonum* L., *Rheum* L., *Ruprechtia* C.A.Mey., *Rumex* L., *Salta* Sanchez and Reveal, *Symmeria* Benth. and *Triplaris* Loefl. [4]–[10]. Of these, *Persicaria*, *Polygonum*, *Rheum* and *Rumex* are more or less cosmopolitan, while the others are more restricted in their range, and a few have disjunct distributions across Australasia and South America as well as Africa and South America (e.g., *Muehlenbeckia* and *Symmeria* respectively). Based on fossil pollen from central Europe, Polygonaceae were estimated to have a minimum age corresponding to the Maastrichtian (approximately 65.5–70.6 Myr) to the Palaeocene (circa 55.8–65.5 Myr) [11], [12].

*Muehlenbeckia* is unique within Polygonaceae because of its amphi-Pacific disjunct distribution pattern. Eighteen species of *Muehlenbeckia* occur in Australasia and nine others are endemic to Central and South America (Table 1). Therefore, *Muehlenbeckia* is a good model to address vicariance and long distance dispersal (LDD) hypotheses for the Southern Hemisphere. The historic biogeography of Southern Hemisphere genera with a similar distribution to that of *Muehlenbeckia*, such as *Araucaria* Juss., *Hebe* Comm. ex Juss., *Nothofagus*, *Podocarpus* L'Hér. ex Pers. and *Weinmannia* L., is often viewed in the context of an ancient Gondwanan association of Antarctica, Australia (including Tasmania), New Caledonia, New Guinea, New Zealand and (southern) South America. Vicariance is a process that leads to speciation through the fragmentation of a widespread ancestral population by physical barriers, such as the breakup of landmasses due to plate tectonic movement. Extant taxa with disjunct distributions therefore, are likely the product of these taxa rafting to their present day localities on the fragmented landmasses that once made up Gondwana. Lineages that occur on constituent Gondwanan landmasses were long thought of as classic examples of vicariant speciation [13]. Recently a paradigm shift has taken place concerning Southern Hemisphere biogeography, and LDD is thought to be at least equally as important for Southern Hemisphere plant group disjunctions [14]–[19]. This shift has occurred mainly because new molecular dating methods applied to Southern Hemisphere lineages have shown that in many cases these groups are much younger than the last physical contact of the vicariant landmasses they occur on [13], [16]–[21]. Because *Muehlenbeckia* has a so-called Gondwanan distribution (Table 1), we address the question of whether vicariant speciation is likely for this Southern Hemisphere group. Mainly, our goal is to report the first age estimates for major clades of Polygonaceae, which are based on Bayesian phylogenetic analyses of molecular data calibrated with fossils. Hypotheses for the biogeographic history of Southern Hemisphere groups such as *Muehlenbeckia* are also discussed.

**Table 1:** The extant distribution of species of *Muehlenbeckia*.

Species	Extant distribution	Species	Extant distribution
**1.** ***Muehlenbeckia adpressa*** (Labill.) Meisn.	Australia incl. Tasmania	**15.** *Muehlenbeckia monticola* Pulle	New Guinea
**2.** *Muehlenbeckia andina* Brandbyge	Bolivia, Ecuador, Peru	**16.** *Muehlenbeckia nummularia* H.Gross	Peru
**3.** ***Muehlenbeckia arnhemica*** K.L.Wilson & R.O.Makinson, ined.	Australia	**17.** ***Muehlenbeckia platyclada*** (F.Muell.) Meisn.	New Guinea, Solomon Islands
**4.** ***Muehlenbeckia astonii*** Petrie	New Zealand	**18.** *Muehlenbeckia polybotrya* Meisn.	Australia
**5.** ***Muehlenbeckia australis*** (G..Forst.) Meisn.	New Zealand, Norfolk Island	**19.** ***Muehlenbeckia rhyticarya*** F.Muell. ex Benth.	Australia
**6.** ***Muehlenbeckia axillaris*** (Hook.f.) Endl.	Australia incl. Tasmania, New Zealand	**20.** *Muehlenbeckia sagittifolia* (Ortega) Meisn.	Argentina, Bolivia, Brazil, Paraguay, Uruguay
**7.** ***Muehlenbeckia complexa*** (A.Cunn.) Meisn.	New Zealand, Lord Howe Island	**21.** ***Muehlenbeckia tamnifolia*** (Kunth) Meisn.	Argentina, Bolivia, Colombia, Ecuador, El Salvador, Guatemala, Honduras, Mexico, Panama, Peru, Venezuela
**8.** ***Muehlenbeckia costata*** K.L.Wilson & R.O.Makinson, ined.	Australia	**22.** ***Muehlenbeckia tiliifolia*** Wedd.	Bolivia, Colombia, Ecuador, Peru
**9.** ***Muehlenbeckia diclina*** (F.Muell) F.Muell.	Australia	**23.** *Muehlenbeckia triloba* Danser	Australia
**10.** ***Muehlenbeckia ephedroides*** Hook.f.	New Zealand	**24.** ***Muehlenbeckia tuggeranong*** Mallinson	Australia
**11.** *Muehlenbeckia fruticulosa* (Walp.) Standl.	Bolivia, Chile, Peru	**25.** ***Muehlenbeckia urubambensis*** Brandbyge	Peru
**12.** ***Muehlenbeckia gracillima*** Meisn.	Australia	**26.** ***Muehlenbeckia volcanica*** (Benth.) Endl.	Costa Rica, Bolivia, Colombia, Ecuador, Guatemala, Mexico, Peru
**13.** ***Muehlenbeckia gunnii*** (Hook.f.) Endl.	Australia incl. Tasmania	**27.** ***Muehlenbeckia zippelii*** (Meisn.) Danser	Australia, New Guinea
**14.** *Muehlenbeckia hastulata* (Smith) I.M.Johnston	Argentina, Chile		

The 19 species of *Muehlenbeckia* analysed in this study are shown in bold font.

## Materials and Methods

### Taxon Sampling

The data set included 81 species of Polygonoideae (with an emphasis on *Muehlenbeckia* and its closest relatives *Fallopia* and *Reynoutria* [22]), Eriogonoideae and Plumbaginaceae, and the latter were used as outgroup. *Afrobrunnichia* and *Symmeria* were not included in this study because their position is labile. Nineteen of the 27 currently recognized species of *Muehlenbeckia* were included in the analyses and are shown in bold font in Table 1. Molecular data were not available for *Muehlenbeckia andina*, *M*. *fruticulosa*, *M*. *hastulata*, *M*. *monticola*, *M*. *nummularia*, *M*. *polybotrya*, *M*. *sagittifolia* and *M*. *triloba*. Species authors are not given in the text, but in Table 1 and Appendix S1.

### Alignment

Molecular data of four gene regions (2678 base pairs) were used including the two chloroplast (cp) genes *mat*K and *ndh*F, one cp intergenic spacer region *trn*L-*trn*F, and the nuclear marker ribosomal ITS. Most sequences used here were generated for previous studies in Polygonaceae [22]–[23] and NCBI accession numbers are given in Appendix S1.

Sequences were aligned with Mafft v.6.717b [24] and option L-INS-i. This strategy assumes that there is one alignable domain that is flanked by difficult to align residues. Each DNA region was aligned individually and poorly aligned regions (characters with more than 50% gaps) were excluded from the analyses with Gblocks 0.91b [25]. Congruence of datasets was tested with the Congruence Among Distance Matrices (Cadm) test by using Kendall's W statistic [26]. We created a smaller dataset that contained only those species available for all four gene regions (35 taxa), because the program can only test congruence of distance matrices of the exact same size. This dataset included species from nearly all tribes (excluding Rumiceae). All aligned regions were concatenated into one dataset, because Cadm indicated congruence among the gene regions and comparison of bootstrap support among individual datasets and combined cp vs. nuclear analyses did not indicate strongly supported conflict. See Appendix S2 in Supporting Information for the alignment file.

### Phylogenetic Analysis–Bayesian Inference

The dataset was analysed with MrBayes v.3.1.2 [27]. Two parallel Bayesian analyses with four chains each and partitioned by DNA region were run for 10 million generations, a sample frequency of 1000 and a burn-in of 25%. Evolutionary models for each DNA region were determined by the Akaike Information Criterion (AIC) [28] with MrAIC v.1.4.3 [29]. Detailed information about the models and priors used is given in Appendix S3. Tracer v.1.5 [30] was used to evaluate mixing of chains and to determine burn-in. Posterior Probabilities (PP) indicate clade support and we define values of 1.00–0.90 as good, 0.89–0.70 as moderate and 0.69–0.50 as weak support.

### Phylogenetic Analysis–Maximum Likelihood

A Maximum Likelihood (ML) phylogeny was inferred with RAxML 7.0.4 sequential version [31] based on the same dataset used for the MrBayes analysis. The partitioned ML analysis was conducted with a general time reversible (GTR) substitution model and 1000 rapid bootstrap replicates [32], with the latter indicating clade support. Likelihood of the final tree was optimized under GAMMA. We consider 100–90% bootstrap support (BS) as good, 89–70% as moderate and 69–50% as low.

### Age Estimates–Fossil Calibration Points

We used seven fossils and a maximum age constraint of 125 Myr on the root of the tree in order to calibrate the Beast analyses. Based on the current consensus for the likely appearance of angiosperms 125 Myr ago [33]–[40], we consider this to be the maximum age for Polygonaceae. Several pollen fossil dates and a leaf fossil of *Muehlenbeckia* from New Zealand are available for Polygonaceae. The publications citing the fossils used for our age calibrations (Table 2) include micrographs of the fossil pollen and illustrations of the leaf fossil, and can therefore be identified as belonging to the clades they were used to calibrate. For example, based on venation pattern and leaf shape, the leaf fossil was determined as similar to *Muehlenbeckia australis* by Pole [41]. To the best of our knowledge, only *Reynoutria* has a similar leaf shape and venation pattern, but it is not native to New Zealand or Australia, which is why we agree with Pole's determination of the leaf fossil as *Muehlenbeckia*. The leaf fossil was dated to 12.7–22.0 Myr in the Miocene and was used to calibrate the age prior for the divergence of *M*. *australis* from its sister species. We use Ogg [42] and Cooper [43] to determine absolute ages of the fossils.

**Table 2:** A compilation of fossils cited for Polygonaceae.

Fossil	Location	Myr–Epoch or Stage	Publication
**A**			
*Muehlenbeckia* sp.	Manuherikia Group, New Zealand	22.0–19.0 (Otaian)–15.0–12.7 (Lillburninan)	[41]
(partial leaf)			
**B**			
*Muehlenbeckia*–like (pollen)	Murray Basin, Australia	39.6 (upper Miocene – Pliocene)	[69]
*Rhoipites muehlenbeckiaformis*			
**C**			
*Polygonum* sect. *Duravia*	Northwest U.S.A.	11.6–5.3 (upper Miocene)	[12], [97]
(pollen)			
**D**			
*Calligonum* sp.	Sahara	5.3–2.6 (Pliocene)	[12], [98]
(pollen)			
**E**			
*Persicaria* sp.	Central Europe	65.5–55.8 (Palaeocene)	[12], [99]–[103]
(pollen)	Assam-India	16.0–11.5 (middle Miocene)	[104]
**F**			
*Coccoloba* sp.	Mexico	11.6–5.3 (upper Miocene)	[12], [105]
(pollen)			
**G**			
*Armeria* sp.	Spain	11.6–5.3 (upper Miocene)	[12], [106]
(pollen)			

**Figure 1:**
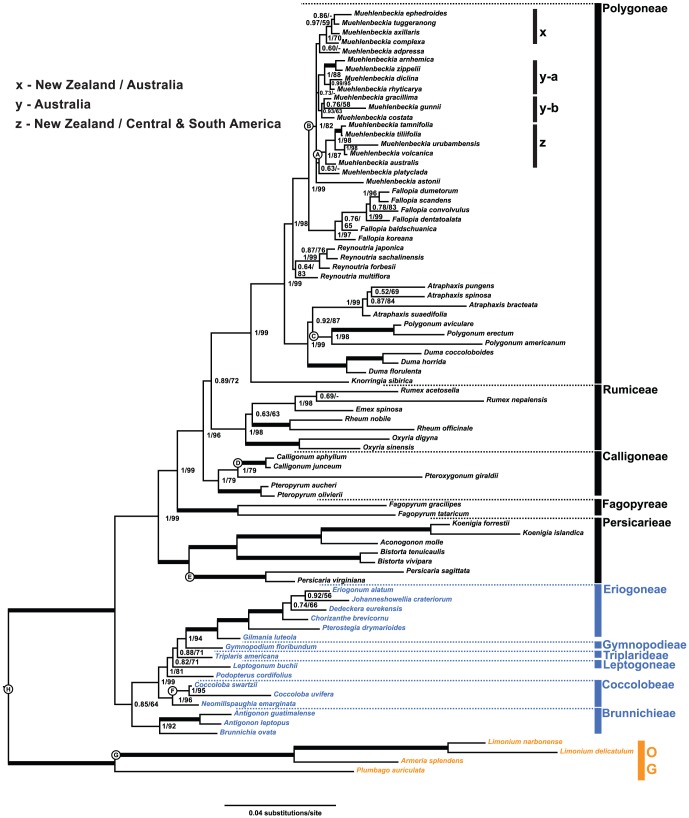
Fifty percent majority rule Bayesian tree. Tree results from a MrBayes analysis of an 81-species dataset including four combined chloroplast and nuclear gene regions (*mat*K, *ndh*F, *trn*L-*trn*F and nrITS). The analysis was run with 10 million generations and a burn-in of 25%. Clade support above 0.49 posterior probability (PP) and 49% bootstrap support (BS) is shown on the tree, and thick branches indicate a support of 1.00 PP/100% BS. Subclades within *Muehlenbeckia* that correspond to the geographical regions shown to the left of the tree are indicated as x, y-a, y-b and z. Polygonoideae are shown in black font, Eriogonoideae are indicated in blue and Plumbaginaceae (outgroup) in orange. The positions of fossil calibration points used in the Beast analyses are indicated by circled letters A–G (Table 3). Letter H indicates the maximum age constraint of 125 million years, which corresponds to the appearance of eudicots in the fossil record.

Million years are abbreviated as (Myr) and absolute ages follow Ogg [42] and Cooper [43]. Letters correspond to fossil position for the calibration scheme indicated in Fig. 1.

### Estimating Divergence Times–Relaxed Molecular Clock Analyses

In order to estimate divergence times within Polygonaceae, relaxed molecular clock analyses [44] were done with Beast 1.6.2 [45]. Because we cannot be certain for how long a clade already existed before a particular fossil find, analyses were performed with uniform and non-uniform priors, which each operate under different assumptions that can impact the age estimates. The uniform and non-uniform priors were used to explore the range of possible ages and to get limits for the oldest and youngest age estimates of each clade. We performed two types of analyses with fossil calibrations: (1) uniform, hard minimum priors (2) exponential/lognormal distribution priors using an exponential distribution for pollen fossils and a lognormal distribution for the leaf fossil. We chose exponential priors for pollen fossils, because pollen data give a good estimate of the first occurrence of a taxon [46], [47], which is reflected in the shape of the exponential distribution [48]. Lognormal priors are better suited when the assumption is reasonable that a clade is older than the fossil find [48]. Because leaf fossils are much more rare than pollen fossils, they may be weak indicators of the exact time a clade occurred [47]. Both exponential and lognormal priors can be calibrated to give the highest probability distribution close to the date of the respective fossil. Uniform priors allow for a higher range of uncertainty in the analysis but tend to estimate older ages than exponential or lognormal priors [40].

For all analyses, we set the maximum tree height to 125 Myr (Table 3). For the analysis using uniform priors, the lower bound was set to the youngest age of the fossil and the upper bound was set to 125 Myr. For the analysis using exponential priors (pollen fossils) the younger fossil ages were used as offset and the mean was set to the older fossil dates. With this option, there is a greater probability for a clade to be older than the oldest fossil age, because a clade has to be present before its fossils accumulate in the stratigraphic record [47], [48]. For the same reason, we set the offset to the lowest and the mean to the upper age value for the lognormal priors (leaf fossil). Where no age range was available (Table 3), we used the given fossil age as offset and set the mean 10% older.

**Table 3:** Dating strategies for the Beast analyses.

	Fossil A	Fossil B	Fossil C	Fossil D	Fossil E	Fossil F	Fossil G
	*Muehlenbeckia*	*Muehlenbeckia*	*Polygonum*	*Calligonum*	*Persicaria*	*Coccoloba*	*Armeria*
	(leaf)	-like (pollen)	(pollen)	(pollen)	(pollen)	(pollen)	(pollen)
**Uniform**	Lower: 12.7	Lower: 39.6	Lower: 5.3	Lower: 2.6	Lower: 55.8	Lower: 5.3	Lower: 5.3
	Upper: 125.0	Upper: 125.0	Upper: 125.0	Upper: 125.0	Upper: 125.0	Upper: 125.0	Upper: 125.0
**Expo/log**	Lognormal	Exponential	Exponential	Exponential	Exponential	Exponential	Exponential
	Mean: 17.0	Mean: 44.0	Mean: 11.6	Mean: 5.3	Mean: 65.5	Mean: 11.6	Mean: 11.6
	Offset: 12.7	Offset: 39.6	Offset: 5.3	Offset: 2.6	Offset: 55.8	Offset: 5.3	Offset: 5.3
	STDV: 1						

Dating strategies used for six Polygonaceae and one Plumbaginaceae (outgroup-G) fossils (details in Table 2) for the analyses with either uniform or exponential/lognormal priors. Prior settings are given in million years and maximum tree height was set to the likely age of eudicots at 125 Myr (see text for citations). Letters assigned to fossils correspond to those shown on the MrBayes tree (Fig. 1).

A Yule prior was used to construct the tree and the ucld.mean was adjusted to a uniform prior of 10–0.000001 to reflect reasonable substitution rates per site for plants (Simon Ho, personal communication). Two independent runs for each prior setting (uniform and exponential/lognormal) were done in Beast with 100 million generations. Further details about parameters and priors are given in Appendix S3. The phylogenetic tree constructed with RAxML was used as a starting tree and all groups for which a fossil was available, and which were supported as monophyletic by the RAxML and MrBayes analyses, were constrained as monophyletic in Beast. Branch lengths of the starting tree were transformed to ages with the nonparametric rate smoothing (NPRS) algorithm [49] implemented in TreeEdit v1.0a10 [50]. Each run was evaluated with Tracer v.1.5 [30] for correct mixing of chains and stable ESS (effective sample size) values and burnin was set to 10%. Both runs were combined with LogCombiner v.1.6.2. [45] and summarized with TreeAnnotator v.1.6.2. [45].

## Results

### Phylogenetic Analyses

The program Gblocks excluded 621 sites for nrITS, 140 for *mat*K, 378 for *ndh*F and 567 for *trn*L-*trn*F (39% of the original alignment). The Cadm test indicated that the individual gene regions are congruent and yielded a Kendal's W statistic of 0.84 (p-value of 0.001), where 0 denotes incongruent data and 1 equals maximum congruency. The Akaike information criterion suggested a general time reversible model (GTR) of sequence evolution with GAMMA distribution of rates across sites (GTRG) for *mat*K and *trn*L-*trn*F, and the addition of invariant sites for *ndh*F, and nrITS (GTRIG). Maximum Likelihood and MrBayes analyses resulted in the same phylogenetic relationships (Fig. 1, tree files in Appendix S4). Results discussed in the text mainly consider the MrBayes analysis, and findings for the ML analysis are shown as bootstrap support values on the Bayesian phylogram in Figure 1.

Polygonaceae are composed of two large clades that correspond to Eriogonoideae and Polygonoideae (Fig. 1). Within Polygonoideae, the addition of several more species of *Atraphaxis* in our study shows for the first time that this clade is sister to *Polygonum* (0.92 PP/87% BS) and that *Duma* is sister to this pair (1.00 PP/99% BS). Subclades formed by species of *Muehlenbeckia* from Australia and New Zealand (clade x), Australia (clades y-a and y-b), and species from New Zealand and Central and South America (clade z) receive good to moderate support. The relationships among these clades are weakly supported though.

With the exception of *M*. *adpressa* and *M*. *tuggeranong*, the Australian species of *Muehlenbeckia* form a clade (y-a plus y-b), which receives some support (0.73 PP/-% BS) in the MrBayes analysis. The well-supported clade y-a (1.00 PP/88% BS) includes the tropical *M*. *arnhemica* from northern Australia plus *M*. *zippelii*, which occurs in north eastern Australia and New Guinea, and another species pair formed by *M*. *diclina* and *M*. *rhyticarya*, which occur in southern Australia and on the East coast respectively (Fig. 1). The second clade of Australian species of *Muehlenbeckia* (y-b) includes the predominantly coastal species *M*. *gunnii* from southern Australia and Tasmania as well as the fire-ephemerals *M*. *costata* and *M*. *gracillima*. Clade y-b receives a support of 0.93 PP/63% BS. *Muehlenbeckia tuggeranong* in clade x, which is a narrow endemic of eastern Australia, is nested among species from New Zealand. The widespread *M*. *adpressa*, which occurs in the southern half of Australia as well as Tasmania, may be sister to clade x, but this relationship receives weak support. Also included in clade x is *M*. *axillaris*, which occurs in Australia, Tasmania and predominantly New Zealand. Clade z contains a well-supported subclade (1.00 PP/87% BS) of Central and South American species as sister to *M*. *australis* from New Zealand. *Muehlenbeckia platyclada* from New Guinea and the Solomon Islands is indicated as sister to clade z by both the MrBayes and ML analyses, but with weak support (0.63 PP/-% BS).

### Age Estimation

**Figure 2:**
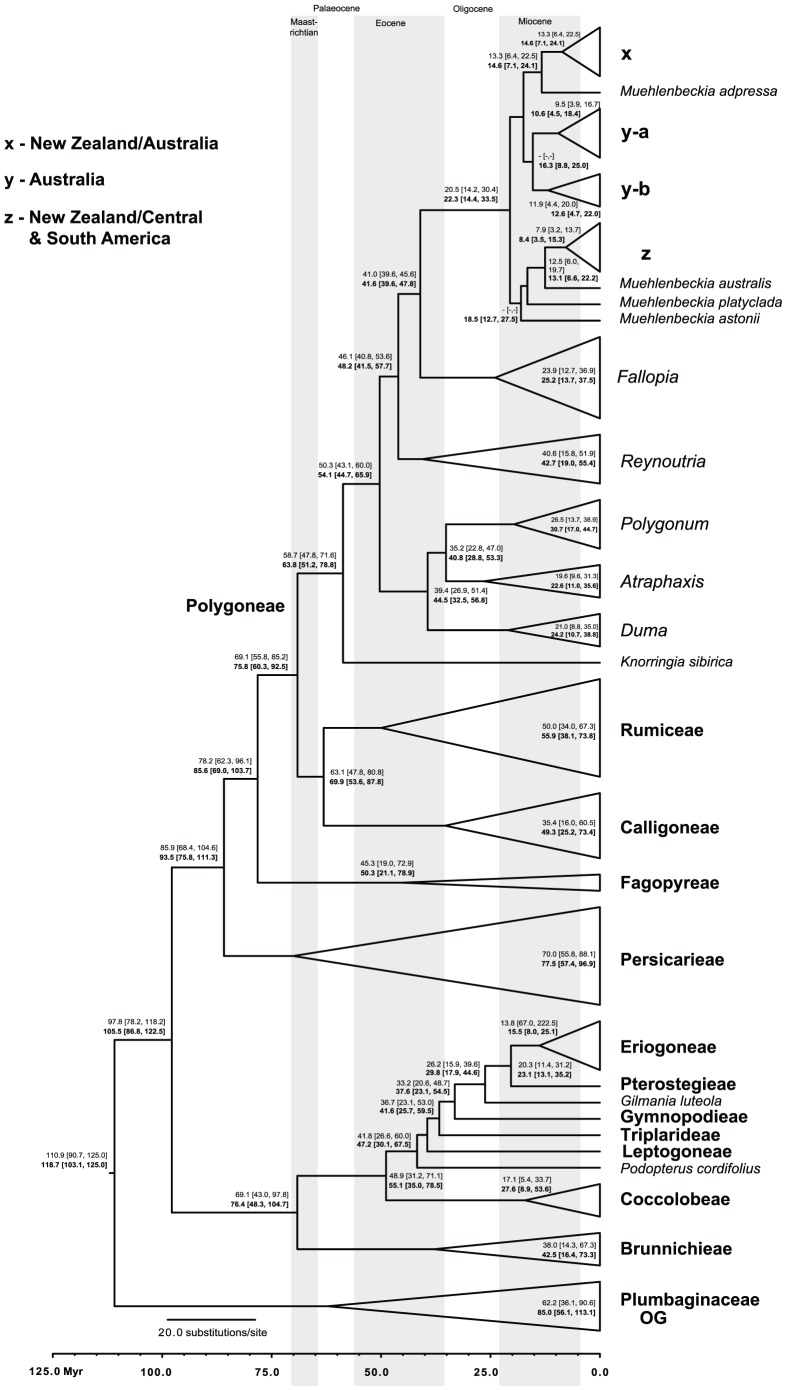
Results from the Beast analyses. Mean age estimates within Polygonaceae from the exponential/lognormal (top, regular font) and uniform (bottom, bold font) prior analyses are shown on the tree from the exponential/lognormal analysis. Highest posterior density (HPD) values (95%) are shown in square brackets. Mean age estimates and HPD values are not shown for nodes below 0.50 posterior probability. The scale axis indicates millions of years, and grey bars correspond to the stratigraphic ages Maastrichtian, Eocene and Miocene, while white bars indicate the Palaeocene and Oligocene. Subclade designations (x, y-a, y-b, z) within *Muehlenbeckia* correspond to clades recovered in the Bayesian analysis shown in Fig. 1 where fossil calibration points and individual species are shown. The corresponding geographic states for clades (x, y-a, y-b, z) are indicated to the left of the tree. Subclade designations for *Muehlenbeckia*, tribes of Polygonaceae and the outgroup are shown in bold font.

The uniform and exponential/lognormal analyses resulted in different age estimates (Fig. 2, Table 4). In all cases, age estimates for the uniform are older than for the exponential/lognormal analysis. Following, we show the youngest and oldest age of the 95% highest posterior density range of both analyses combined in parentheses after the exponential/lognormal and uniform prior mean age estimates.

**Table 4:** Age estimates for selected stem clades of Polygonaceae.

	Uniform in Myr	Exponential/Lognormal in Myr
	Mean (95% HDP)	Mean (95% HDP)
**Polygonaceae**	118.7 (103.1–125.0)	110.9 (90.7–125.0)
**Eriogonoideae**	105.5 (86.8–122.5)	97.8 (78.2–118.2)
Brunnichieae	76.4 (48.3–104.7)	69.1 (43.0–97.8)
Coccolobeae	55.1 (35.0–78.5)	48.9 (31.2–71.1)
Eriogoneae	23.1 (13.1–35.2)	20.3 (11.4–31.2)
Gymnopodieae	37.6 (23.1–54.5)	33.2 (20.6–48.7)
Triplarideae	41.6 (25.7–59.5)	36.7 (23.1–53.0)
**Polygonoideae**	105.5 (86.8–122.5)	97.8 (78.2–118.2)
Persicarieae	93.5 (75.8–111.3)	85.9 (68.4–104.6)
Fagopyreae	85.6 (69.0–103.7)	78.2 (62.3–96.1)
Calligoneae	69.9 (53.6–87.8)	63.1 (47.8–80.8)
Rumiceae	69.9 (53.6–87.8)	63.1 (47.8–80.8)
Polygoneae	75.8 (60.3–92.5)	69.1 (55.8–85.2)
*Duma*	44.5 (32.5–56.8)	39.4 (26.9–51.4)
*Muehlenbeckia*	41.6 (39.6–47.8)	41.0 (39.6–45.6)
(stem clade)		
*Muehlenbeckia*	22.3 (14.4–33.5)	20.5 (14.2–30.4)
(crown clade)		

Mean age estimates based on Beast analyses for selected clades of Polygonaceae given in million years (Myr), rounded to one decimal point and with 95% highest posterior density (HPD) ranges shown in parentheses. The analyses were calibrated with seven fossil dates (see Tables 2 and 3 for fossil ages and calibration scheme, and Fig. 1 for fossil placement on the tree) and run either with uniform or exponential and lognormal distributions for the priors. Root height was constrained to 125 Myr, which likely correspond to the age of eudicots (see text for citations).

With respect to divergence times (Table 4), results of the Beast analyses (Fig. 2, Appendix S4) indicate that the split between Polygonaceae and its sister group Plumbaginaceae is relatively old with 110.9/118.7 (90.7–125.0) Myr as compared to the age of eudicots at 125 Myr. This is also much older than previous ages given for Polygonaceae (55.8–70.6 Myr). The two main clades in the buckwheat family, Eriogonoideae and Polygonoideae split 97.8/105.5 (78.2–122.5) Myr ago and Polygoneae, which include the Southern Hemisphere genera *Duma* and *Muehlenbeckia*, likely diverged 69.1/75.8 (55.8–92.5) Myr ago. The stem node of *Muehlenbeckia* indicates that it diverged from its sister clade in the Eocene with a minimum age of 41.0/41.6 (39.6–47.8) Myr (Fig. 2). Most extant species of *Muehlenbeckia* diversified 20.5/22.3 (14.2–33.5) Myr ago, and the crown clade of the Central and South American group diversified 7.9/8.4 (3.2–15.3) Myr ago. The stem node of the Australian endemic *Duma* diverged around 39.4/44.5 (26.9–56.8) Myr ago, which is a similar time frame as for *Muehlenbeckia*, and *Duma*'s crown clade diversified around 21.0/24.2 (8.8–38.8) Myr ago.

## Discussion

### Phylogenetic Relationships

Results of the current MrBayes and ML analysis (Fig. 1) of one nr (ITS) and three cp (*mat*K, *ndh*F and *trn*L-*trn*F) markers for the most part show the same results for the evolutionary relationships among and within all genera included when compared to the ML and Maximum Parsimony analyses of other studies (e.g., [1], [22], [23], [51], [52]). Since the evolutionary relationships among clades and the placement of genera within Polygonaceae are congruent across these studies, these results will not be reiterated here and only differences are discussed briefly. For example, the placement of *Gilmania luteola* with respect to *Pterostegia drymarioides* differs from Kempton's recent study [53] on Eriogonoideae. In Kempton's analysis, *G*. *luteola* is placed as sister to all other Eriogoneae, and Pterostegieae (including *P*. *drymarioides* and *Harfordia macroptera*) is sister to that clade with both relationships well supported. In our study and in previous analyses *G*. *luteola* branches before *P*. *drymarioides* and Eriogoneae with good support (1.00 PP/100% BS) and Burke and Sanchez [52] include *Pterostegia* in Eriogoneae. Since Kempton's [53] taxon sampling for Eriogonoideae and in particular Eriogoneae is much denser, we defer to her results. Furthermore, in Polygonoideae, the addition of more data for *Atraphaxis* resolved its position as sister to *Polygonum* (0.92 PP/87% BS), which is a novel result.

While most subclades within *Muehlenbeckia* receive good to moderate support, the relationships among these clades are not clear from the MrBayes and ML analyses, and more data are necessary to clarify this. For the most part, relationships within *Muehlenbeckia* are consistent with results from previous studies (e.g., [22]). In Schuster et al.'s [22] study, most species of *Muehlenbeckia* that occur in Australia formed a clade (except *M*. *axillaris* and *M*. *tuggeranong*), albeit with weak bootstrap support. The current MrBayes analysis shows similar results, except that *M*. *adpressa* may be included in clade x along with *M*. *tuggeranong*, *M*. *axillaris* and other species from New Zealand. Clade y-a includes a species pair of the tropical *M*. *arnhemica* from northern Australia and *M*. *zippelii* from north eastern Australia and New Guinea, and another species pair formed by *M*. *diclina* from southern Australia and *M*. *rhyticarya* from the East coast. The relationship of these two sister pairs shows a pattern observed in other groups of Australian plants, such as *Eucalyptus* L′Hér and *Jacksonia* Rees [54], [55] as well as birds including *Melithreptus* honeyeaters [56] and fairy wrens [57]. There appears to be a deep split between a Monsoon group from the Northern Territory and an East/South Coast group, which may have once been separated by the Carpentarian barrier [54]–[57]. The second clade (y-b) formed by Australian species of *Muehlenbeckia* in the MrBayes analysis includes *M*. *gunnii* from southern Australia and Tasmania as well as *M*. *costata* and *M*. *gracillima* from the East Coast. Clade z is always recovered with good support and is composed of a well-supported subclade formed by all Central and South American species sampled, which is sister to *M*. *australis* from New Zealand.

### Age Estimates and Fossil Calibrations

These are the first age estimates for clades of the buckwheat family Polygonaceae. Our findings are based on dating methods using a relaxed molecular clock model calibrated with one leaf fossil of *Muehlenbeckia* and six pollen fossils of this genus and other Polygonaceae as implemented in Beast. Using fossils for calibration is not an easy task (e.g., [58] and references therein), but it remains the best dating method currently available when care is taken with the calibration process [59], [60]. Our results will allow for further hypothesis testing in a historical biogeographic context, although there are relatively large time span errors (Fig. S1).

Results from this study indicate that Polygonaceae likely diverged much earlier than previously thought (55.8–70.6 Myr ago) with estimated mean ages of 110.9 Myr for the exponential/lognormal and 118.7 Myr for the uniform analyses and comparatively early with respect to other eudicots (125 Myr ago). Taking the 95% highest posterior density values into account, the age estimates range from 90.7–125 Myr (Fig. 2). Given that eudicots are thought to have emerged approximately 125 Myr ago, this is relatively old for a group in the superasterids (including Asteridae, Caryophyllales and Santalales [61]). Eudicot (tricolpate) pollen appears in the fossil record about 125 Myr ago [33] and this date is well accepted based on the presence of monocolpate pollen and spores in earlier stratigraphic layers [35]. It stands to reason though that eudicots could be older than 125 Myr, because they likely originated before the massive and abrupt appearance of tricolpate pollen in the fossil record. To our knowledge, no other studies discuss the age of Polygonaceae specifically, but several authors give estimates for the age of Caryophyllales. Ages of 99–102 Myr for crown Caryophyllales [35], 94.2–94.5 and 110.7–111.3 Myr for their crown and stem ages respectively [36], 104–111 Myr [62] and approximately 101 Myr [61] are given. Different data, analytic and fossil calibration approaches utilizing one to many fossils were used for these studies and they are therefore not necessarily comparable, but their results indicate a range of 94–111 Myr for the emergence of Caryophyllales.

The fossils used to calibrate the trees in these studies are for the most part macrofossils. Only one macrofossil, a *Muehlenbeckia*-like leaf has so far been reported for Polygonaceae [41], while several more calibration points are available when fossil pollen is taken into account. Thornhill et al. [47] argue that pollen fossils have several advantages over macrofossils due to the durability of sporopollenin and because they are stratigraphically and temporally vastly more abundant. Therefore, the probability of fossil pollen indicating a date closer to the actual origin of a group is higher. A weak point for pollen fossils is the limited availability of morphological characteristics in some groups [40 and references therein], [47]. In Polygonaceae however, pollen morphology is a character of potentially great phylogenetic value [11], [63]–[65]. For example, in *Polygonum* the ektexine clearly differentiates the four recognized sections in the genus. *Persicaria*, which had been included in *Polygonum* until recent molecular analyses showed that it is not closely related to this group [66]–[68], has a rather different pollen type as well [11], [63]–[65]. It is also important to note that one character that supports the segregation of *Duma* from *Muehlenbeckia* [22] is that they have a completely different pollen morphology as evidenced by Scanning Electron Microscopy data. While *Muehlenbeckia* has a punctate-striate pollen morphology, *Duma* has a faveolate pollen surface with micro-spinules [69], [70]. This supports the inclusion of fossil pollen data in our analyses. Using pollen fossils allows for more calibration points, which estimates rate heterogeneity among lineages better and should result in more accurate age estimates [47]. Thornhill et al.'s [47] results indicate that calibrations with additional fossil pollen dates yield older estimated ages compared to analyses dated with macrofossils alone, and this might explain our comparatively old age estimates for Polygonaceae with respect to previous analyses of Caryophyllales.

In addition, in our results, age estimates are consistently older for the uniform than for the exponential/lognormal analysis. Other authors [40] using a similar calibration scheme also found that exponential priors resulted in younger ages than analyses using uniform priors. This is not unexpected, because in our calibration scheme the exponential/lognormal priors gave a much smaller probability to the maximum age of 125 Myr than the uniform priors. In our exponential/lognormal prior calibration, the mean probability distribution was at the older age boundary of the fossil, whereas in the uniform calibration the probability for ages ranging from the maximum to the minimum age was the same. This was done to give a higher probability to ages older than the fossil find date for the exponential/lognormal analyses (see Material and Methods for more explanation). Setting the mean age to a date closer to the lower age boundary could have potentially resulted in slightly younger age estimates for the exponential/lognormal analyses. However, the time span between offset (younger fossil age) and mean (older date) overall only differed between 2.7 and 9.7 Myr (Table 3), so it is unlikely that this would have resulted in a considerably younger age of Polygonaceae. Interestingly, overall variation of ages was similar in the exponential/lognormal and uniform analyses (33.4 vs. 33.1 Myr respectively for clades shown in Table 4 and Fig. S1).

### Biogeographic Hypotheses

With respect to the historical biogeography of Polygonaceae, Schuster et al. [51] noted that the family might have its origin in Africa, because the African *Symmeria* and *Afrobrunnichia* likely are sister to all other members of the family [23], [52]. The difficulty with testing this hypothesis is that the position of *Afrobrunnichia* is uncertain and strongly varies with taxon sampling and genetic markers used as does the position of *Symmeria* when *Afrobrunnichia* is excluded. Therefore, we decided to exclude these two species from our analyses. Until more data for these important African species are available, we can only develop hypotheses about the historical biogeography of Polygonaceae.

For Polygonaceae we here propose a working hypothesis, which involves either an African or a Gondwanan ancestor that gave rise to an American and Caribbean lineage (Eriogonoideae) and a second, widespread lineage that mostly occurs in the Northern Hemisphere (Polygonoideae). The question is whether diversification of the two main clades Eriogonoideae and Polygonoideae can be explained by vicariance or LDD. If the African *Afrobrunnichia* and *Symmeria* with a disjunct distribution in Africa and South America are indeed sister to the rest of the family vicariance seems somewhat plausible for Eriogonoideae, because the age estimates of 97.8/105.5 (78.2–122.5) Myr (Table 4) fit the time frame for the separation of South America from Africa 119–105 Myr ago [71]. Clades within Eriogonoideae indicate a complex pattern of dispersal events between Central and South America, the Caribbean as well as western and eastern North America. The disjunction of the South American and African *Symmeria* will require further testing to say more about vicariance or LDD patterns of Eriogonoideae.

Polygonoideae may have an even more complex history, because they include several large clades with a worldwide distribution (*Persicaria*, *Polygonum* and Rumiceae). Within Polygonoideae, *Knorringia sibirica* from Central Asia and Yunnan is always indicated as sister to all other members of Polygoneae (Fig. 1). Within Polygoneae, the split between the mainly Australasian *Muehlenbeckia* and its closest relative *Fallopia* is dated at 41.0/41.6 (39.6–47.8) Myr. Most extant species of *Fallopia* occur in temperate Asia (mainly China, Japan and Korea) although some species are widespread due to anthropogenic factors [72]. *Reynoutria*, another genus from temperate Asia, is sister to *Fallopia* + *Muehlenbeckia*. It is plausible that the ancestor of *Muehlenbeckia* could have spread to Australia and/or New Zealand from temperate Asia, because there is evidence for exchange of taxa between Asia and Australia in the Miocene [54], [73], [74]. The presence of *Muehlenbeckia* in Oceania may be explained by stepping-stone dispersal of its ancestor from Asia (maybe via New Guinea). It should be noted that the extant *Fallopia* and *Reynoutria* are not native to Australia and New Zealand and that *Muehlenbeckia* is not extant in temperate Asia.

Alternatively, one could argue that the origin of *Muehlenbeckia* was a vicariant event, in which its ancestor rafted on a Gondwanan fragment such as India or Australia, because India made contact with Asia approximately 43 Myr ago and Australia is thought to be isolated only since 35–28 Myr ago [71], [75]. However, by definition, a vicariance explanation for the diversification of *Muehlenbeckia* implies that the clades formed by species of this group which are found in New Zealand (clade x), Australia (clades y-a, y-b) and South America (subgroup of clade z) were present on all of these constituent land masses before the breakup of Gondwana 95–30 Myr ago [76]. This is unlikely, since diversification of the crown clade of *Muehlenbeckia* is estimated at 20.5/22.3 (14.2–33.5) Myr (Fig. 2, Table 4), which is too young to satisfy the vicariance hypothesis. At 14.4–33.5 Myr, the 95% highest posterior density values of the uniform prior analysis for *Muehlenbeckia* (Fig. 2) is close to the breakup age of Gondwana, but all clades within *Muehlenbeckia* with disjunct distributions across e.g. New Zealand and South America are too young for vicariance.

For example, the chronogram (Fig. 2) shows evidence of LDD from New Zealand to Australia in *Muehlenbeckia*'s clade x. *Muehlenbeckia tuggeranong*, which only occurs in Australia, is nested within clade x among species from New Zealand. Vicariance is unlikely, because crown clade x is estimated to be 13.3/14.6 (6.4–24.1) Myr old and Australia has been isolated since 35–28 Myr while New Zealand has been cut off since approximately 80–56 Myr. Long distance dispersal is likely also the most parsimonious explanation for the diversification of *Muehlenbeckia*, because the South American species of *Muehlenbeckia* are sister to *M*. *australis*, which is native to New Zealand and Norfolk Island in clade z. The age estimates for the split of *M*. *australis* and the South American clade at 12.5/13.1 (6.0–22.2) Myr is younger than the isolation ages of South America and New Zealand. South America has been isolated since 30 Myr [71], [76] and New Zealand is thought to be isolated since 55.8 Myr [40], [77], though dates around 80 Myr are more commonly found in the literature (e.g., [71], [75]).

The date for the diversification of the Central and South American subclade in clade z (*M. tamnifolia*, *M. tiliifolia*, *M. urubambensis*, *M. volcanica*) at 7.9/8.4 (3.2–15.3) Myr correlates well with age estimates for a second uplift of the Eastern Cordilleras of the Northern and Central Andes [78], [79]. Mountain building may also have influenced climatic and edaphic factors, since high mountain ranges create a barrier to precipitation [78]. Climatic and edaphic factors, landslides and erosion could have created a mosaic of microhabitats that afforded new possibilities for diversification [80], [81] as is thought to have happened in the species-rich Cape Floristic Province in South Africa [82]. Radiation events during the uplift of the Andes have also been reported in other groups such as Chloranthaceae [83], Ericaceae [84], Fabaceae [85], Rubiaceae [78], Lepidoptera [86] and hummingbirds [87].

Most diversification events in *Muehlenbeckia* occurred after 20.5/22.3 (14.2–33.5) Myr ago, which correlates with the aridification and cooling of Australia in the Miocene [88]–[91]. Aridification may have resulted in an increase in the frequency of bushfires. Several of the Australian species of *Muehlenbeckia* (in clades y-a and y-b) are adapted to fire [22]. For example, the fire-ephemeral *M*. *diclina* grows in scleromorphic mallee, a habitat characterized by stands of *Eucalyptus* L'Hér., *Acacia* Mill. and *Triodia* R.Br., which are adapted to burns. The fire-adapted species of *Muehlenbeckia* are difficult to classify as facultative or obligate seeders, because they usually senesce before another burn. However, they do respond strongly to fire cues for germination (Peter Clarke, personal communication). In contrast, the flora of New Zealand has few fire-adapted species and the Australian species that belong to the mixed Australian/New Zealand clade (x), such as *M*. *tuggeranong* and *M*. *axillaris* are also not adapted to fire. Radiation of Australian groups in the Miocene is observed frequently, and the crown clade of *Duma* also diversified within this time frame around 21.0/24.2 (8.8–38.8) Myr ago. *Allocasuarina* L.A.S. Johnson, *Banksia* L.f. [75], some Elaeocarpaceae Juss. [74], *Eucalyptus* [92] and some scleromorphic groups of Fabaceae Lindl. [93] also radiated during the Miocene in Australia. Similar adaptations to disturbance that may be caused by fire have occurred in other ecosystems, such as the South African fynbos, the chaparral in California, the Chilean matorral and the South American cerrado [37], [38], [94]–[96].

To summarize, because the sister genera of *Muehlenbeckia* mainly occur in temperate Asia, which has never been considered part of Gondwana, and because clades of *Muehlenbeckia* with disjunct distributions across e.g. New Zealand and South America are younger than when these landmasses broke apart, LDD rather than vicariance is likely the main driver for diversification within this group. The crown clade of *Muehlenbeckia* diversified 20.5/22.3 (14.2–33.5) Myr ago, and this is younger than the isolation dates of Australia (35–28 Myr), Antarctica (32–30 Myr), New Zealand (80–55 Myr) and South America (32–30 Myr). Our age estimates for Polygonaceae and clades such as *Muehlenbeckia* are a starting point for further testing of its phylogeny in a biogeographic context. This will give more insights about the origin of Polygonaceae and the diversification of specific clades within this diverse and widespread family.

## Supporting Information

Figure S1 **Comparison of age estimate variation (difference between highest and lowest 95% highest posterior density values) for selected clades of Polygonaceae from the Beast analyses using either exponential/lognormal or uniform priors.**
(EPS)

Appendix S1 **NCBI accession numbers and voucher information for sequence data used in this study.**
(DOC)

Appendix S2 **Concatenated alignment file for the four analysed gene regions,** ***ndh*** **F,** ***mat*** **K,** ***trn*** **L-** ***trn*** **F and nrITS.**
(NEX)

Appendix S3 **Input files for the 1) exponential/lognormal and 2) uniform prior analyses in Beast for divergence time estimation of Polygonaceae.**
(XML)

Appendix S4 **Tree files resulting from the 1) Maximum Likelihood, 2) MrBayes, 3) Beast exponential/lognormal prior and 4) Beast uniform prior analyses.**
(TXT)
