# Supplementary material for: Age Estimates for the Buckwheat Family Polygonaceae Based on Sequence Data Calibrated by Fossils and with a Focus on the Amphi-Pacific Muehlenbeckia
Source: PLoS One. 2013 Apr 5;8(4):e61261. doi: 10.1371/journal.pone.0061261 (PMC3621405; doi:10.1371/journal.pone.0061261)
Supplement: Appendix S1 — NCBI accession numbers and voucher information for sequence data used in this study. (DOC) [file pone.0061261.s002.doc]

**Appendix S1** Sequence data used in this study, which was generated for an analysis done by Schuster et al. (2011a). Data for *Atraphaxis pungens* and *A*. suaedifolia have been added to that dataset. GenBank accession numbers for the gene regions used are given in the following order: nrITS, *mat*K, *ndh*F, *trn*L-*trn*F, while a dash (–) corresponds to no available data.

*POLYGONACEAE*: ***Aconogonon molle*** (D. Don) H. Hara, EF653687, GQ206190, GQ206271, EF653790; ***Antigonon guatimalense*** Meisn., FJ154461, FJ154491, FJ154503, –; ***Antigonon leptopus*** Hook. & Arn., FJ154462, EF437988, EF438027, –; ***Atraphaxis bracteata*** Losink., JN187099, –, –, EU109601; ***Atraphaxis pungens*** (M.Bieb.) Jaub. & Spach, JN187100, –, EU840370, EU840538; ***Atraphaxis spinosa*** L., FJ154463, EF437989, EF438028, JF831296; ***Atraphaxis suaedifolia*** Jaub. & Spach, AB542773, –, –, AB542784; ***Bistorta tenuicaulis*** (Bisset & S. Moore) Nakai, GQ206240, –, GQ206274, –; ***Bistorta* *vivipara*** (L.) Delarbre var. ***vivipara***, DQ372903, –, –, EU024776; ***Brunnichia ovata*** (Walter) Shinners, FJ154465, AY042561, EF438029, –; ***Calligonum aphyllum*** (Pall.) Gürke, –, GQ206192, GQ206275, JQ009290; ***Calligonum* *junceum*** (Fisch. & C.A. Mey.) Litv., GQ206243, GQ206194, GQ206277, EU109590; ***Chorizanthe* *brevicornu*** Torr. **var. *brevicornu***, FJ154466, EF437991, EF438030, –; ***Coccoloba swartzii*** Meisn., –, EF437995, EF438034, –; ***Coccoloba uvifera*** (L.) L., GQ206246, EF437996, –, AJ312249; ***Dedeckera eurekensis*** Reveal & J.T.Howell, FJ154470, EF437997, EF438036, –; ***Duma*** ***coccoloboides*** (J.M. Black) T.M. Schust., JF831204, JF831264, JF831243, JF831297; ***Duma*** ***florulenta*** (Meisn.) T.M. Schust., JF831205, JF831265, JF831244, JF831298; ***Duma* *horrida*** (H. Gross) T.M. Schust. **subsp. *horrida***, JF831206, JF831232, JF831245, JF831299; ***Emex spinosa*** (L.) Campd., FJ154471, AY042582, EF438037, –; ***Eriogonum alatum*** Torr. **var. *alatum***, FJ154472, EF437998, EF438038, –; ***Fagopyrum gracilipes*** (Hemsl.) Dammer ex Diels, AB000332, AB026314, –, EU024787; ***Fagopyrum tataricum* subsp. *pontanini*** (L.) Gaertn., AB000339, AB089712, –, EU109606; ***Fallopia baldschuanica*** (Regel) Holub, AF040063, –, –, EU024779; ***Fallopia convolvulus*** (L.) Á. Löve, AF040064, EU024770, –, EU024782; ***Fallopia dentatoalata*** (F. Schmidt) Holub, AF040066, EU024769, –, EU024775; ***Fallopia dumetorum*** (L.) Holub, AF040068, AM503813, –, EU024785; ***Fallopia koreana*** B.U. Oh & J.G. Kim, AF040061, –, –, AF189740; ***Fallopia scandens*** (L.) Holub, AF040069, –, –, EF653785; ***Gilmania luteola*** (Coville) Coville, GQ206250, EF438010, EF438049, –; ***Gymnopodium floribundum*** Rolfe, GQ206251, GQ206197, GQ206282, –; ***Johanneshowellia crateriorum*** Reveal, GQ206252, EF438011, EF438050, –; ***Knorringia sibirica*** (Laxm.) Tzvelev, GQ206253, EU024771, GQ206284, EU024789; ***Koenigia forrestii*** (Diels) Měsíček & Soják**,** GQ206254, EF438012, EF438051, FJ821779; ***Koenigia islandica*** L., DQ406625, –, –, EF653789; ***Leptogonum buchii*** Urb., GQ206256, GQ206199, GQ206285, –; ***Muehlenbeckia adpressa*** (Labill.) Meisn., JF831207, JF831233, JF831246, JF831300; ***Muehlenbeckia arnhemica*** K.L. Wilson & R.O. Makinson, ined. (Muehlenbeckia sp. Mt Brockman) K.L. Wilson & R.O. Makinson ms, JF831221, JF831234, JF831247, JF831301; ***Muehlenbeckia astonii*** Petrie, EF635479, JF831266, JF831248, JF831302; ***Muehlenbeckia australis*** (G. Forst.) Meisn., JF831208, JF831267, JF831249, JF831303; ***Muehlenbeckia axillaris*** (Hook. f.) Endl., JF831209, JF831268, JF831250, JF831304; ***Muehlenbeckia complexa*** (A. Cunn.) Meisn., AF040076, GQ206200, JF831251, JF831305; ***Muehlenbeckia costata*** K.L. Wilson & R.O. Makinson, ined. (Muehlenbeckia sp. Mt Norman) K.L. Wilson & R.O. Makinson ms, JF831210, JF831269, JF831252, JF831306; ***Muehlenbeckia diclina* subsp. *stenophylla* (Muehlenbeckia subsp. Gippsland)** (F. Muell.) K.L. Wilson & R.O. Makinson, JF831211, JF831235, JF831253, JF831308; ***Muehlenbeckia ephedroides*** Hook. f., JF831212, JF831236, JF831254, JF831307; ***Muehlenbeckia gracillima*** Meisn., JF831213, JF831237, JF831255, JF831309; ***Muehlenbeckia gunnii*** (Hook. f.) Endl., - JF831214, JF831238, JF831256, JF831310; ***Muehlenbeckia platyclada*** (F. Muell.) Meisn., AF189738, JF831239, JF831257, JF831311; ***Muehlenbeckia rhyticarya*** F. Muell. ex Benth., AF189739, **–**, **–**, JF831312; ***Muehlenbeckia tamnifolia*** (Kunth) Meisn., FJ154473, FJ154499, FJ154511, JF831313; ***Muehlenbeckia tiliifolia*** Wedd., JF831215, JF831270, JF831258, JF831314; ***Muehlenbeckia tuggeranong*** Mallinson, JF831216, JF831271, JF831259, JF831315; ***Muehlenbeckia urubambensis*** Brandbyge, JF831217, JF831240, JF831260, JF831316; ***Muehlenbeckia volcanica*** (Benth.) Endl., JF831218, JF831241, JF831261, JF831317; ***Muehlenbeckia zippelii*** (Meisn.) Danser, JF831219, JF831242, JF831262, JF831318; ***Neomillspaughia emarginata*** (H. Gross) S.F. Blake, GQ206257, GQ206201, GQ206287, –; ***Oxyria digyna*** (L.) Hill, FJ154474, FJ154500, FJ154512, AY566466; ***Oxyria sinensis*** Hemsl., GQ206258, EF438013, EF438053, –; ***Persicaria sagittata*** (L.) H.Gross, FJ154476, EF438018, GQ206288, EF653799; ***Persicaria virginiana*** (L.) Gaertn., EU410358, EF438019, EF438058, EF653801; ***Podopterus cordifolius*** Rose & Standl., FJ154479, FJ154494, FJ154505, –; ***Polygonum americanum*** (Fisch. & C.A. Mey.) T.M. Schust. & Reveal [*Polygonella americana* (Fisch. & C.A. Mey.) Small], GQ206259, GQ206202, GQ206289, –; ***Polygonum aviculare*** L., FJ493492, EF438020, EF438059, FJ627271; ***Polygonum erectum*** L., GQ206260, GQ206203, –, –; ***Pteropyrum aucheri*** Jaub. & Spach, AB542780, GQ206205, GQ206292, AB542791; ***Pteropyrum olivierii*** Jaub. & Spach, AB542782, –, GQ206293, AB542793; ***Pterostegia drymarioides*** Fisch. & C.A. Mey., GQ206263, GQ206206, GQ206294, –; ***Pteroxygonum giraldii*** Dammer & Diles, DQ406627, GQ206207, GQ206295, EU402464; ***Reynoutria forbesii*** (Hance) T. Yamaz. [*Fallopia forbesii* (Hance) Yonekura & Ohashi], AF040072, –, –, –; ***Reynoutria japonica*** Houtt. [*Fallopia japonica* (Houtt.) Ronse Decr.], AF040070, EU024772, EF438048, EU024786; ***Reynoutria*** ***multiflora*** (Thunb.) Moldenke [*Fallopia multiflora* (Thunb.) Czerep.], AF040053, EF153684, –, EU402461; ***Reynoutria*** ***sachalinensis*** (F. Schmidt) Nakai [*Fallopia sachalinensis* (F. Schmidt) Ronse Decr.], AF040073, EF438009, JF831263, JF831320; ***Rheum nobile*** Hook. f. & Thomson, GQ206264, –, EF438060, AY566465; ***Rheum officinale*** Baill., FJ503007, AB115684, –, AF303431; ***Rumex acetosella*** L., AF189730, EF438022, GQ206298, GQ245413; ***Rumex nepalensis*** Spreng., AF338219, –, –, EU326091; ***Triplaris americana*** L., FJ154486, AY042668, FJ154508, AJ312251.

*PLUMBAGINACEAE*: ***Armeria splendens*** Webb, AY444093, –, –, AJ391316; ***Limonium delicatulum*** Kuntze, AJ222851, –, –, AJ391324; ***Limonium narbonense*** Mill., AJ222838, AF204853, –, AJ391327; ***Plumbago auriculata*** Lam., JF831220, **–**, **–**, JF831319.
